# The Impact of Ambulance and Patient Diversion on Crowdedness of Multiple Emergency Departments in a Region

**DOI:** 10.1371/journal.pone.0144227

**Published:** 2015-12-11

**Authors:** Chung-Yao Kao, Jhen-Ci Yang, Chih-Hao Lin

**Affiliations:** 1 Department of Electrical Engineering, National Sun Yat-Sen University, Kaohsiung, Taiwan; 2 Department of Emergency Medicine, National Cheng Kung University Hospital, College of Medicine, National Cheng Kung University, Tainan, Taiwan; Johns Hopkins Bloomberg School of Public Health, UNITED STATES

## Abstract

Emergency department (ED) overcrowding threatens healthcare quality. Ambulance diversion (AD) may relieve ED overcrowding; however, diverting patients from an overcrowded ED will load neighboring EDs with more patients and may result in regional overcrowding. The purpose of this study was to evaluate the impact of different diversion strategies on the crowdedness of multiple EDs in a region. The importance of regional coordination was also explored. A queuing model for patient flow was utilized to develop a computer program for simulating AD among EDs in a region. Key parameters, including patient arrival rates, percentages of patients of different acuity levels, percentage of patients transported by ambulance, and total resources of EDs, were assigned based on real data. The crowdedness indices of each ED and the regional crowdedness index were assessed to evaluate the effectiveness of various AD strategies. Diverting patients equally to all other EDs in a region is better than diverting patients only to EDs with more resources. The effect of diverting all ambulance-transported patients is similar to that of diverting only low-acuity patients. To minimize regional crowdedness, ambulatory patients should be sent to proper EDs when AD is initiated. Based on a queuing model with parameters calibrated by real data, patient flows of EDs in a region were simulated by a computer program. From a regional point of view, randomly diverting ambulatory patients provides almost no benefit. With regards to minimizing the crowdedness of the whole region, the most promising strategy is to divert *all* patients equally to *all* other EDs that are not already crowded. This result implies that communication and coordination among regional hospitals are crucial to relieve overall crowdedness. A regional coordination center may prioritize AD strategies to optimize ED utility.

## Introduction

Emergency department (ED) overcrowding threatens healthcare quality and is becoming a worldwide problem [1]. Conceptual models of ED management have enhanced the understanding of ED overcrowding [2–5]. Managing the input, throughput, and output processes of EDs can relieve ED overcrowding [6–9]. Ambulance diversion (AD) reduces ED patient input and, thus, may relieve overcrowding [10], especially because ambulance-transported patients are generally sicker and may utilize more ED resources [11,12].

However, diverting patients from an overcrowded ED loads neighboring EDs with more patients and, thus, may result in overcrowding of neighboring EDs. The implementation of AD by any single ED may actually worsen regional emergency health care. The effectiveness of AD policies should be quantitatively evaluated and should be tailored for local practices [13–15].

The difficulty of evaluating AD effectiveness in real situations motivates the use of computer simulation [16]. For one single ED, computer programs for predicting episodes of AD [17] and forecasting ED crowding status [18,19] have been developed based on established models of emergency medical services systems [20,21] and EDs [5,13,22–26]. However, the optimal AD policy for regional health care is unclear.

In an earlier work, we developed a simulation model to explore the effectiveness of different diversion strategies on the crowdedness of single emergency department [27]. The purpose of this study was to evaluate the impact of different ambulance and patient diversion strategies on relieving the crowdedness of several EDs in a region. We also addressed the importance of regional coordination via computer simulation.

## Materials and Methods

### The queuing model for an ED

The queuing model used in this study was developed recently by CH Lin et al. [27] It consists of a set of theoretical probability distributions governing patient flow [28,29]. The structure of the model conforms to the conceptual input-throughput-output framework of ED operations proposed by BR Asplin et al. [5], reflecting care processes that substantially contribute to ED overcrowding. For more details regarding this model, please refer to CH Lin et al. [27] and the references therein.

### The concept of EDs in a region

**Fig 1:**
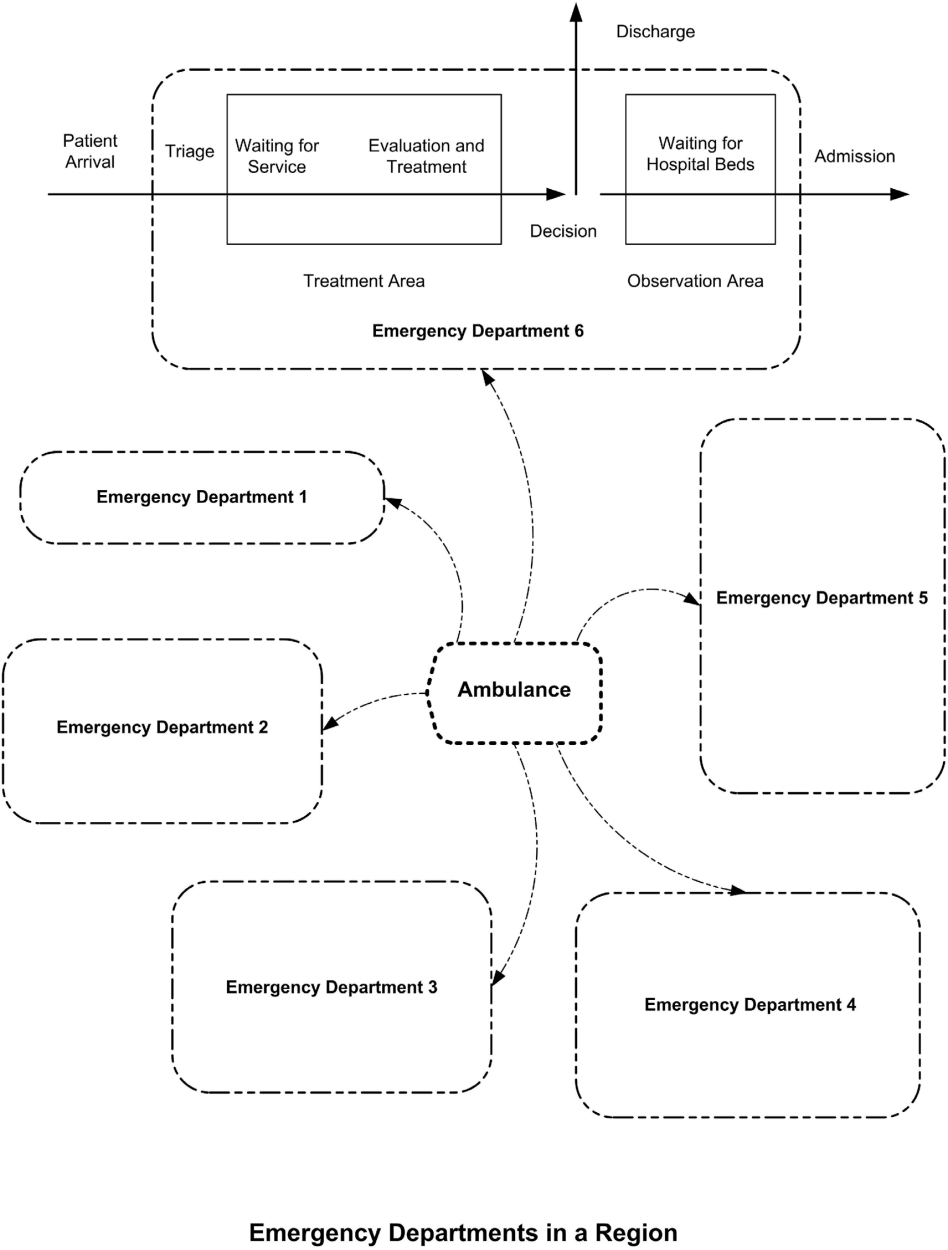
The concept of emergency departments in a region.

The concept of EDs in a region is illustrated in Fig 1. Ambulances may transport patients to either EDs. Each ED has different amount of resources, which is symbolically represented by their different sizes in Fig 1. The conceptual framework of ED operations is shown in the ED 6 in Fig 1.

### The Crowdedness Index and the Regional Crowdedness Index

The *crowdedness index* (CI) of an ED is defined as “the current loading of an ED” compared to “the full capacity of an ED”. The current loading of an ED is defined as the medical resources currently occupied. While the full capacity of an ED is defined as the maximal loading of that ED if all beds in the treatment and observation areas are fully occupied and the patient acuity mix is at its long run average. For more details regarding the definition of CI, please refer to CH Lin et al. [27] and the references therein.

The *regional crowdedness index* (RCI) is defined as “sum of the current loading of all EDs in the region” compared to “sum of full capacity of all EDs in the region”.

### Diversion rules

All AD strategies in this study used CI as the only indicator for ongoing diversion; in other words, AD was implemented when the CI of the ED exceeded a certain threshold.

There are three types of patient-blocking rules: all AD (A-AD), high-acuity AD (H-AD), and low-acuity AD (L-AD). A-AD means that all patients transported by ambulance are diverted. H-AD means severe patients are diverted, that is, only patients with high acuity level of one or two are diverted. L-AD means mild patients are diverted, that is, only patients with low acuity levels of three to five are diverted. With regards to patient safety, diverting ambulance-transported patients with high acuity levels while accepting patients with low acuity levels (H-AD) is generally unacceptable in most communities [27]. Thus we only explored the A-AD rule and the L-AD rule in the simulations.

Once AD is implemented, patients are diverted for a certain period of time, which is referred to as the “AD segment”. At the end of an AD segment, the criterion for AD is reassessed, and if the criterion is still satisfied, another AD segment is initiated, unless the total allowable AD duration per day has been reached. The length of the AD segment and the total allowable AD duration per day is regulated by a community-based consensus in real situations. In all of our simulations, the length of the AD segment and the total allowable AD duration per day were 2 hours and 24 hours, respectively.

### Outcome measures

We measured the CI of each ED and the RCI of the region to evaluate the effectiveness and the impact of each diversion strategy.

### Discrete event simulations

This queuing model is implemented in MATLAB language. For the study of each ED strategy, every scenario was simulated 1,000 times to collect data for statistical analysis. The simulator follows the usual discrete event simulation engine logic [29]. For more details, please refer to CH Lin et al. [27] and the references therein.

### Parameter settings in our simulations

In our simulation studies, patient data from the EDs of six hospitals in the Tainan metropolitan region were used to derive the values of some key parameters of the queuing model. Table 1 shows the distributions of ED patients in the six hospitals in this region in October 2012.

**Table 1:** Distribution of Patients Seeking Emergency Medical Care from Six Hospitals in the Tainan Metropolitan Region in October 2012.

Hospitals	AL1	AL2	AL3	AL4	AL5	Total
ED1 (TH)	21	47	551	512	10	1141
ED2 (KGH)	32	94	1575	1057	175	2933
ED3 (SLH)	126	367	2844	651	80	4068
ED4 (TMH)	119	362	3387	1409	275	5552
ED5 (NCKUH)	165	671	3794	2784	44	7458
ED6 (CMH)	200	1714	8940	1264	11	12129
**Total**	**663**	**3255**	**21091**	**7677**	**595**	**33281**

Note: Abbreviations: AL, acuity level; ED, emergency department; TH, Tainan Hospital; KGH, Kuo General Hospital; SLH, Sin-lau Hospital; TMH, Tainan Municipal Hospital; NCKUH, National Cheng Kung University Hospital; and CMH, Chi Mei Hospital; From the data, the total number of ED patients in one month is 33,281. By this number, the patient arrival rate of our simulation is set to be 44 per hour; According to how these 33,281 patients are distributed to the six hospitals, in our simulations the probabilities of an ED patient received by the six hospitals before AD is implemented are set to be (0.0343, 0.0881, 0.1222, 0.1668, 0.2241, 0.3645) for ED1 to ED6, respectively; According to how these 33,281 patients are distributed to the five acuity levels, we set the probabilities of an ED patient triaged with acuity one to five to be 0.02, 0.08, 0.59, 0.28, and 0.03, respectively.

In our simulation, we assumed 20% of patients are transported by ambulance and that 80% of patients are ambulatory [30]. This assumption conforms to the observations of the daily operations of most EDs in Taiwan. Among the ambulance patients, we assumed that 9.96% are acuity level one, 24% are acuity level two, 44.04% are acuity level three, 20% are acuity level four, and 2% are acuity level five. Among the ambulatory patients, the corresponding assumed percentages are 0.01%, 4%, 62.74%, 30%, and 3.25%, respectively. These settings reflect the patient distributions shown in Table 1 and the fact that patients transported by ambulance are generally sicker than patients going to the EDs on their own accord.

We assumed that patients with acuity levels of one, two, three, four, and five consume 16, 8, 4, 2, and 1 unit(s) of medical resources, respectively, when receiving emergency treatment. This amount is halved when the patient is placed in the observation area. This parameter was derived from observations of ED5. This parameter, together with the parameters of patient arrival rate and patient distributions (to each hospital and each acuity level) allowed us to determine the full capacities of the EDs, which are 36, 91, 127, 173, 232, and 377 units for ED1, ED2, ED3, ED4, ED5, and ED6, respectively. These numbers were calculated using the following formula and rounding to the nearest whole integer:

$$\text{Full}\;\text{capacity}\;\text{of}\;\text{an}\;\text{ED}=\lambda \times 6\times p_{ED}\times \sum_{i=1}^{5}p_{i}\kappa_{i}$$

where λ = patient arrival rate = 44 persons/hour, *p*_ED_ = probability of patient being received by the ED, *p*_*i*_ = probability of a patient received by the ED being of acuity level *i*, and κ_*i*_ = medical resources consumed by an acuity level *i* patient.

Average treatment time, probabilities of a patient being admitted, inpatient bed availability, and other parameters commonly utilized in all of our simulation studies are summarized in Table 2.

**Table 2:** The Parameter Values Utilized in All Our Simulation Studies.

Total MR	ED1 = 36, ED2 = 91, ED3 = 127, ED4 = 173, ED5 = 232, ED6 = 377 (units)
MR consumed by a patient in treatment	AL1 = 16, AL2 = 8, AL3 = 4, AL4 = 2, AL5 = 1 (units)
MR consumed by a patient in observation	AL1 = 8, AL2 = 4, AL3 = 2, AL4 = 1, AL5 = 0.5 (units)
Average treatment time	AL1 = 4, AL2 = 3, AL3 = 2, AL4 = 1, AL5 = 0.5 (hours)
Variation of treatment time	AL1 = 15, AL2 = 9.6, AL3 = 5.4, AL4 = 1.35, AL5 = 0.15 (hours)
Probability of being admitted after treatment	AL1 = 0.9, AL2 = 0.7, AL3 = 0.5, AL2 = 0.3, AL1 = 0.1
Hospital bed availability	1 bed becomes available every 0.5 hour
Patient arrival rate (hourly average)	ED1 = 1.51, ED2 = 3.88, ED3 = 5.37, ED4 = 7.34, ED5 = 9.86, ED6 = 16.04, Total = 44 (persons per hour)
Transportation of patients	Ambulance-transported patients = 20% of the total patients; Ambulatory patients = 80% of the total patients
Acuity distribution of ambulance patients	AL1 = 9.96, AL2 = 24, AL3 = 44.04, AL4 = 20, AL5 = 2 (%)
Acuity distribution of ambulatory patients	AL1 = 0.01, AL2 = 4, AL3 = 62.74, AL4 = 30, AL5 = 3.25 (%)

Note: Abbreviations: ED, emergency department; AL, acuity level; MR, medical resource; The average treatment time, the probabilities of a patient being admitted, and the inpatient bed availability are derived from clinical and administrative observations of the ED5.

### Ethics

This research was using computer simulation and adhered to the appropriate reporting guidelines and community standards for data availability. The data used in the simulation were accessible to public and contained no personally identifiable information. This research was thus exempted from review of Institutional Review Board.

## Results

Using our simulation studies, we attempted to answer the following questions:

### Question I

When the CI of an ED reaches the critical value of 1.0 and AD is initiated, patients are typically diverted to EDs that have more medical resources. To efficiently utilize regional resources, should all ambulance-transported patients or only ambulance-transported patients with low acuities be diverted?

### Question II

When AD is initiated, should the ambulance-transported patients be diverted to EDs with more medical resources or be diverted to any other ED in the region?

### Question III

Would giving EDs the option of diverting ambulatory patients make a significant difference in relieving ED crowdedness? What patient diversion strategy would result in the optimal usage of all medical resources in the region?

The answers to these three questions may be inferred from the results of the following AD strategy studies based on the proposed simulation model.

### AD strategy study I

The first study evaluates the impact of two patient-blocking rules, A-AD and L-AD, on relieving ED overcrowding. For this study, we assume the following patient diversion rules:

#### Rule 1

Patients are *equally* diverted only to *EDs with more medical resources* (i.e., patients are diverted to “larger” EDs).

By this rule, ED1 is allowed to divert its patients to ED2-ED6 (with a probability of diversion to each ED of 0.2), but ED2 is only allowed to divert its patients to ED3-ED6 (with a probability of diversion to each ED of 0.25), and so on. ED6 is the “largest” ED in the region and, thus, is not allowed to divert patients regardless its own crowdedness status. Moreover, a patient already diverted from one ED to another is not allowed to be “re-diverted”.

When the CI reaches the critical value of 1.0, the ED may initiate A-AD and divert all ambulance-transported patients (strategy A-AD), or it may initiate L-AD and divert only ambulance-transported patients with low acuity levels (strategy L-AD).

**Fig 2:**
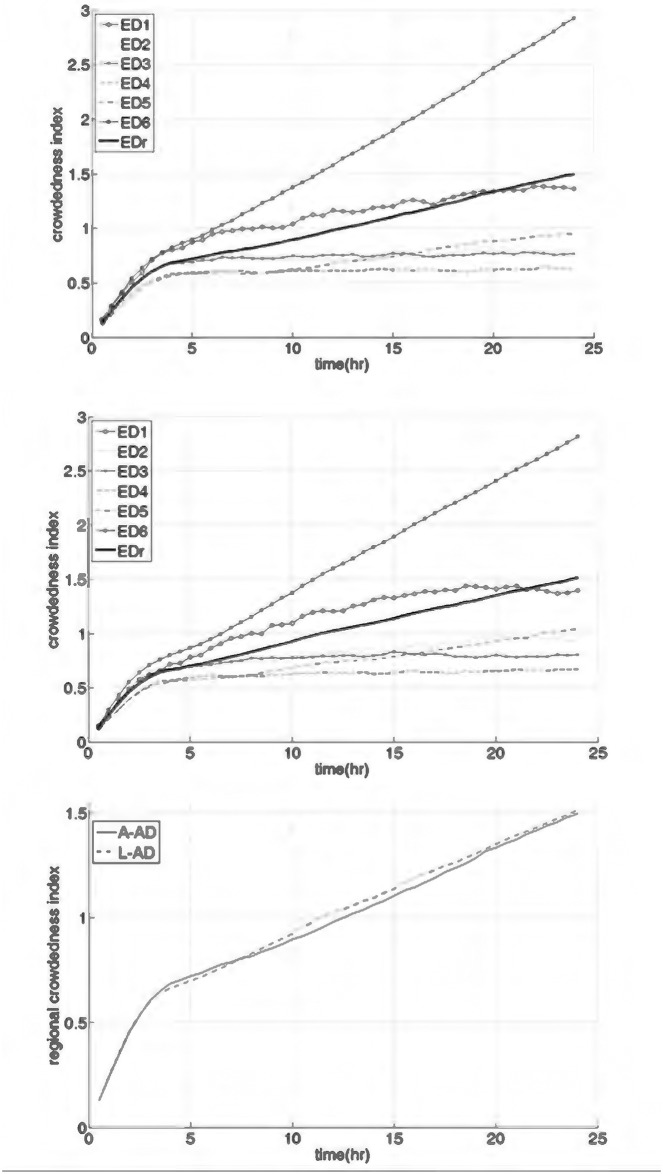
Simulation results of ambulance diversion (AD) strategy: Study I. (a) The crowdedness indices (CIs) of ED1 to ED6 and the regional crowdedness index (represented as EDr) throughout a day resulted from implementing A-AD. (b) The CIs of ED1 to ED6 and the EDr throughout a day resulted from implementing L-AD. (c) The EDr resulted from the two AD strategies.

The simulation results are shown in Fig 2. Fig 2(a) shows the CIs of the six EDs and the RCI throughout a single day while using the A-AD strategy, while Fig 2(b) shows the respective results for the L-AD strategy. The outcomes of the two different strategies were very similar. By either strategy, ED6, which is the “largest” ED in the region, accepts all diverted ambulance-transported patients and becomes excessively overcrowded. The CIs of ED2 to ED5 are below 1.0 throughout the day, while the CI of ED1 is between 1.0 and 1.5. This result indicates that ED1 to ED5 are operated under a non-crowded to mildly crowded status.

The CIs of ED1 to ED5 under the A-AD strategy are slightly smaller than those under the L-AD strategy. This is understandable considering that ambulance-transported patients amount to only 20% of all ED patients. Fig 2(c) shows the insignificance of the difference between the two strategies by comparing the RCIs for the two strategies. The RCI for the A-AD strategy is very slightly lower than that for the L-AD strategy.

### AD strategy study II

The results of study I indicate that when patients are diverted from an ED with fewer resources to EDs with more resources, the ED with the most resources tends to be severely overloaded. Because the A-AD and L-AD strategies produced similar results in study I, in the second study, only the A-AD strategy was used. In this study, we considered two other AD rules in addition to **Rule 1**:

#### Rule 2

Patients are *equally* diverted to *any other* ED. By this rule, ED3 may divert patients to ED1 (with less resources) or ED5 (with more resources). Moreover, all EDs must receive any patient diverted from other EDs, regardless of its own crowdedness and diversion status; that is, no "re-diversion" is allowed.

#### Rule 3

Patients are *equally* diverted only to *EDs that are not in the diversion status* (i.e., patients are diverted to “not crowded” EDs). If all EDs request diversion, then no patient will be diverted.

**Fig 3:**
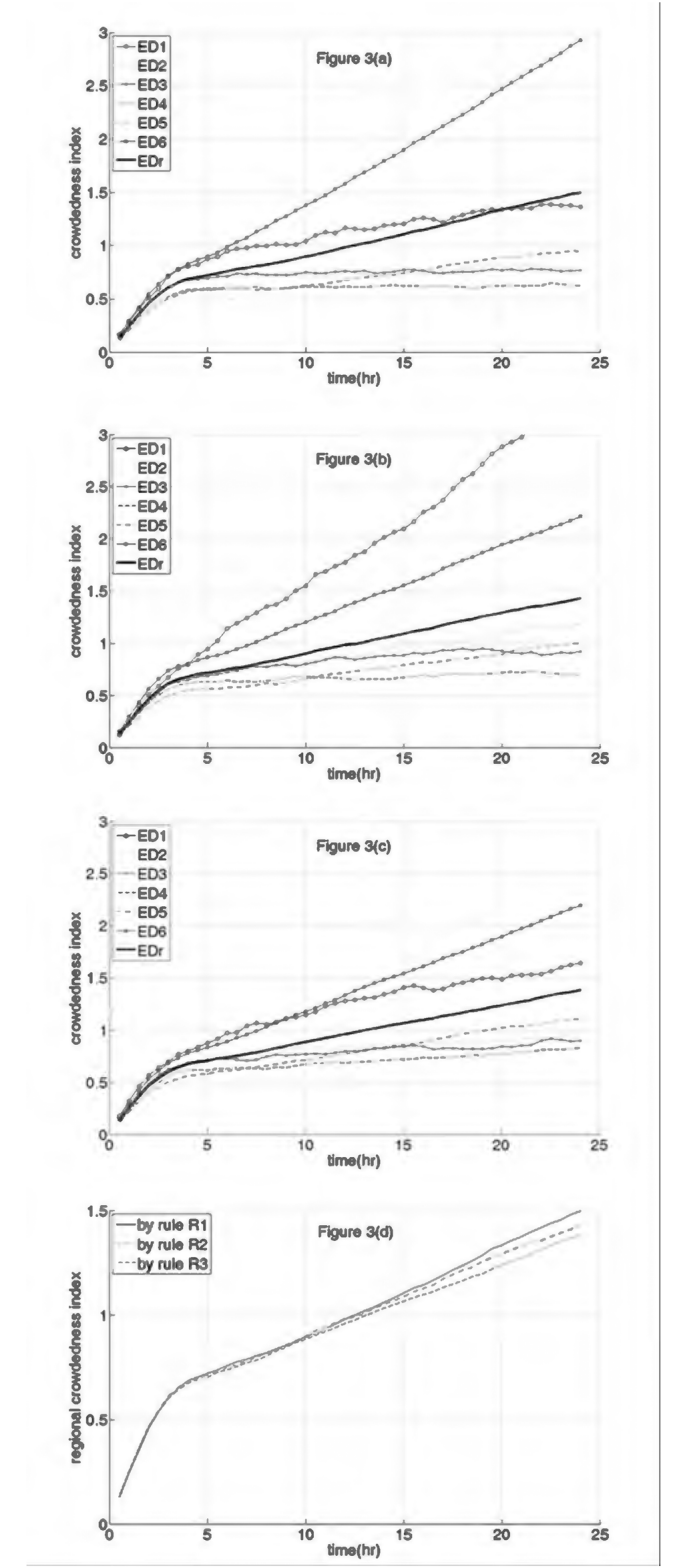
Simulation results of ambulance diversion (AD) strategy: Study II. (a) The crowdedness indices (CIs) of ED1 to ED6 and the regional crowdedness index (represented as EDr) throughout a day resulted from implementing patient diversion rule 1. (b) The CIs of ED1 to ED6 and the EDr throughout a day resulted from implementing patient diversion rule 2. (c) The CIs of ED1 to ED6 and the EDr throughout a day resulted from implementing patient diversion rule 3. (d) The EDr resulted from the three AD strategies.

The results of this simulation are shown in Fig 3. Note that Fig 3(a) using Rule 1 (with A-AD) is exactly the same as Fig 2(a). When following Rule 1, ED6 became excessively overcrowded because it accepted all diverted ambulance-transported patients.

Fig 3(b) shows the simulation results following Rule 2. ED1, which has the least resources in the region, becomes excessively overcrowded. A comparison of Fig 3(b) and 3(a) shows that ED6 becomes less crowded when following Rule 2 than when following Rule 1, but its CI is still over 1.5 for almost half of the day of the simulation.

Fig 3(c) shows the simulation results following Rule 3. A comparison of Fig 3(c) and 3(a) shows that the CIs of ED1 to ED5 when following Rule 3 are only slightly higher than those by Rule 1. Meanwhile, the CI of ED6 when following Rule 3 is slightly lower than that when following Rule 2 and is much lower than when following Rule 1.

The RCIs observed when following Rules 1, 2, and 3 are compared in Fig 3(d), and the RCI observed when following Rule 3 is the smallest.

### AD strategy study III

The results of study II indicate that AD following Rule 3 is most effective, but the improvement in RCI by that method is not significant. In the third study, we explored the effect of diverting ambulatory patients. In this study, we compared **Rule 3** (i.e., ambulance-transported patients are equally diverted to all “not crowded” EDs and ambulatory patients are not diverted) with the following two rules:

#### Rule 4

Ambulance-transported patients are diverted as in **Rule 3.** Ambulatory patients are diverted with no advice as to which ED to attend. Thus, the diverted ambulatory patients may go to *any other EDs with equal probabilities*. In other words, the ambulatory patients are diverted according to **Rule 2.**

#### Rule 5

Ambulance-transported patients are diverted as in **Rule 3.** Ambulatory patients are also instructed to go to *EDs that are not in the diversion status*. Thus, the diverted ambulatory patients may go to *“not crowded” EDs with equal probabilities*. In other words, both ambulance-transported and ambulatory patients are diverted according to **Rule 3.**

**Fig 4:**
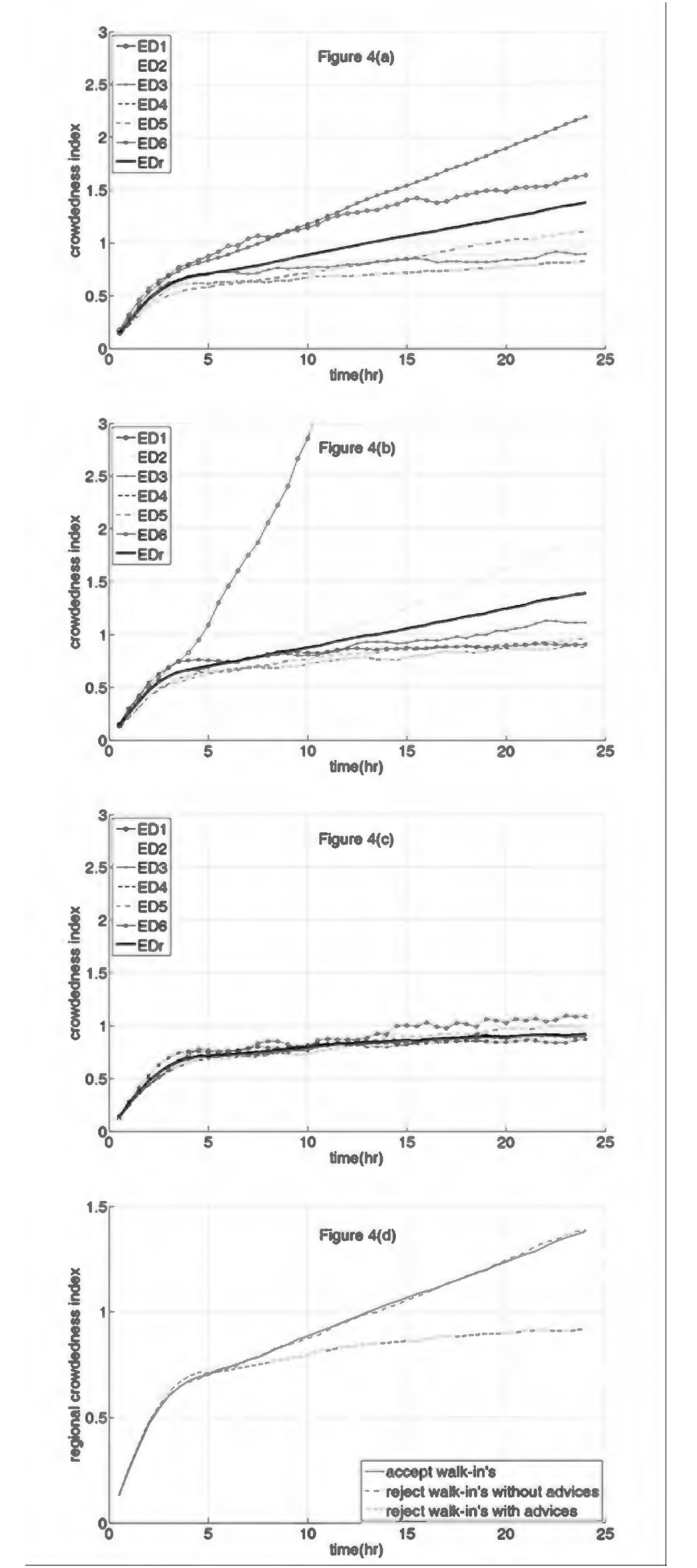
Simulation results of ambulance diversion strategy: Study III. (a) The crowdedness indices (CIs) of ED1 to ED6 and the regional crowdedness index (represented as EDr) throughout a day resulted from the strategy of accepting ambulatory patients. (b) The CIs of ED1 to ED6 and the EDr throughout a day resulted from the strategy of diverting ambulatory patients without giving advices. (c) The CIs of ED1 to ED6 and the EDr throughout a day resulted from the strategy of diverting ambulatory patients with appropriate advices. (d) The EDr resulted from the three patient diversion strategies.

The simulation results are shown in Fig 4. Note that Fig 4(a) using Rule 3 is exactly the same as Fig 3(c). Fig 4(b) and 4(c) show the results when following Rule 4 and Rule 5, respectively. A comparison of Fig 4(a) with Fig 4(b) and 4(c) indicates that there are some fundamental differences when EDs divert ambulatory patients in addition to ambulance-transported patients. This is because ambulatory patients account for 80% of all ED patients.

Fig 4(b) shows that Rule 4 results in ED1 becoming excessively overcrowded within half of a day. Among the other five EDs, ED2 appears to be significantly more crowded than the others, while ED3 is more crowded than ED4, ED5, and ED6.

However, the CIs of ED2-ED5 in Fig 4(c) remain below 1.0 throughout the day, while the CI of ED1 increases to just slightly above 1.0 after 15 hours of the implementation of diversion.

The RCIs obtained by following Rules 3, 4, and 5 are compared in Fig 4(d). The RCI curves obtained by following Rules 3 and 4 are similar to each other, and in comparison, the RCI curve obtained by following Rule 5 is significantly lower, and the RCI is maintained below 1.0 throughout the day.

## Discussion

Three sets of patient diversion strategies were evaluated via computer simulations based on a simplified ED model that represents the operations of various EDs using statistical processes. While the simplified ED model and the assumptions made on the statistical processes may not entirely reflect real ED operations, these processes can nevertheless capture the average dynamics of patient flow in EDs and are widely acceptable. The key parameters of the model, including patient arrival rates, percentages of patients of different acuity levels, percentage of patients arriving by ambulance, and the total resources of the EDs, were assigned based on real data from six hospitals in the Tainan metropolitan region.

Study I evaluated the impacts of diverting all ambulance-transported patients (A-AD) and diverting only low-acuity ambulance-transported patients (L-AD). According to the simulation results, A-AD is slightly more effective than L-AD, as expected, but this difference was insignificant. This result occurred because ambulance patients accounted for only 20% of all patients in our model, and apparently, ambulatory patients contribute substantially to ED crowdedness. The results of study I suggest that when ambulance-transported patients account for a small fraction of the total patients in the EDs of a region, diverting only ambulance-transported patients has limited impact on relieving ED crowdedness. Moreover, diverting ambulance-transported patients of any acuity level has a similar outcome to only diverting those with low acuity levels.

Assuming EDs are only allowed to divert ambulance-transported patients, study II evaluated the impact of three different diversion rules on the relief of overcrowding in several EDs in a region. The simulation results indicate that diverting patients to EDs with more resources causes severe crowdedness in the largest ED in the region, while diverting patients equally to the other EDs tends to severely overcrowd the smallest ED in a region. Neither of these two strategies requires coordination among EDs, and clearly, neither is optimal. Diverting patients to “less crowded” EDs was expected to produce a better outcome, and this was confirmed by comparing the respective RCIs.

Study II demonstrated that the RCI is smaller when patients are diverted only to EDs that are not in the diversion status (Rule 3), comparing to diverting patients to any other ED (Rule 2). The phenomena may be due to some EDs having idle capacity during periods that other EDs are full since they were not recipients of diverted patients. It also hints that regional ED utility could be optimal when diversion status of each ED is accessible to public.

Study III assessed the effectiveness of the diversion of both ambulatory and ambulance-transported patients. Based on the observed RCIs, we found that the outcome of arbitrarily diverting ambulatory patients is very similar to that of not diverting them because, in our simulations, the patients were unable to be re-diverted. Hence, any patient that was diverted by one ED will consequently be accepted by another ED, regardless of whether the ED was overcrowded or not.

The results of study III further suggest that the EDs with less resource tend to suffer when ambulatory patients were diverted arbitrarily. To improve ED efficiency, it is essential that EDs divert ambulatory patients appropriately. When ambulatory patients were diverted to EDs that were not in the diversion status, the RCI curve dropped significantly and was maintained below a certain level throughout the day. The simulation showed that all EDs operated below or at their full capacities and that no overcrowded situations occurred throughout a day.

Therefore, from a regional point of view, simply diverting ambulatory patients provides almost no benefit. However, if ambulatory patients are properly diverted to the EDs that are less crowded, the crowdedness of the EDs in the region improves significantly. This result implies that communication and coordination among regional hospitals are crucial to relieve overall crowdedness. If such a mechanism is viable, our simulation results indicate that it would optimize the utilization of emergency medical resources in a region.

### Limitations

To reduce the complexity of the simulations, we intentionally ignored the time required for certain ED operations, such as triaging patients, cleaning the treatment area, and any administrative processes. Furthermore, many aspects of ED management are grouped into the treatment process. These include laboratory and radiological examinations, administration of medications, pending consultations, explanations to obtain patient consents for certain procedures and treatments, medical education prior to discharge, etc.

In the simulations, we assumed that hospital beds opened at a fixed rate. Although this assumption does not reflect the reality of hospital operations, it nevertheless does not largely change the outcomes in our simulations. In our simulations, we determined that ED input is the main contributor to crowding, as we deliberately overloaded ED capacity by setting an excessive patient influx. To assess ED output as the key source of crowding, it would be necessary to choose a more realistic statistical process to represent hospital bed availability.

Our model may accommodate EDs of different sizes. The impact of AD strategies may vary according to the number of hospitals in a single community as well as the treatment capabilities and capacities of each ED. These are all parameters that can be adjusted in our simulation program.

## Conclusion

Based on a queuing model with parameters calibrated by real data, patient flows of six EDs in a region were simulated by a computer program. The results indicate that with regards to minimizing the crowdedness of EDs in the whole region, the best strategy is to divert all ambulance-transported patients and ambulatory patients to the EDs that are not already crowded. A regional coordination center may prioritize AD strategies to optimize ED utility. The implication of the results of this study should be tailored and be validated in real practice.
